# Coffee Consumption, Serum Copeptin, and Selected Echocardiographic Parameters in Patients with ADPKD: A Cross-Sectional Study

**DOI:** 10.3390/jcm15207943

**Published:** 2026-10-14

**Authors:** Bennur Esen, Ahmet Engin Atay, Ferdi Karagoz, Irfan Sahin

**Affiliations:** 1 Department of Internal Medicine and Nephrology, Bağcılar Training and Research Hospital, University of Health Sciences, 34200 Istanbul, Türkiye; 2 Department of Internal Medicine, Bağcılar Training and Research Hospital, University of Health Sciences, 34200 Istanbul, Türkiye; 3 Department of Cardiology, Bağcılar Training and Research Hospital, University of Health Sciences, 34200 Istanbul, Türkiye

**Keywords:** autosomal dominant polycystic kidney disease, coffee, copeptin, vasopressin, diastolic function, echocardiography, e′ velocity

## Abstract

**Background/Objectives:** Coffee consumption has been suggested to influence arginine vasopressin-related pathways and cardiovascular function. However, its relationships with serum copeptin and cardiac parameters in patients with autosomal dominant polycystic kidney disease (ADPKD) remain unclear. This study aimed to investigate the association between habitual coffee consumption and serum copeptin concentrations and selected echocardiographic parameters in patients with ADPKD. **Methods:** In this cross-sectional study, 73 patients with ADPKD were classified as coffee consumers (*n* = 35) or non-consumers (*n* = 38). Serum copeptin measurements were available for 62 patients. Coffee consumption was assessed using a standardized questionnaire and expressed as grams of dry coffee per month. Multivariable models were used to assess associations with log-transformed copeptin, mitral annular e′ velocity, and E/e′ ratio. **Results:** Serum copeptin levels were lower among coffee consumers than non-consumers (median, 263.51 vs. 332.77 ng/L; *p* = 0.013). After adjustment, coffee consumption was associated with lower log-transformed copeptin levels (B = −0.263; *p* = 0.047), although this association was not statistically significant when using heteroskedasticity-consistent (HC3) robust standard errors (*p* = 0.074). Coffee consumers had higher mitral annular e′ velocity (8.61 vs. 5.86 cm/s; *p* < 0.001), which remained significant after adjustment (B = 2.531; *p* < 0.001), and a lower E/e′ ratio (B = −4.548; *p* = 0.007). **Conclusions:** In this ADPKD cohort, coffee consumption was associated with lower serum copeptin levels, higher mitral annular e′ velocity, and lower E/e′ ratio. These findings raise the hypothesis that habitual coffee consumption may be associated with vasopressin–copeptin system activity and diastolic myocardial relaxation in patients with ADPKD. However, the copeptin association was not robust to HC3 analysis, and the echocardiographic findings should be considered exploratory.

## 1. Introduction

Autosomal dominant polycystic kidney disease (ADPKD) is the most common inherited monogenic kidney disease and is characterized by progressive cyst formation, kidney enlargement, and gradual deterioration of renal function [1]. Beyond pathogenic variants in the *PKD1* and *PKD2* genes, arginine vasopressin (AVP)-mediated signaling plays an important role in disease progression [2]. Activation of the vasopressin V2 receptor stimulates intracellular cyclic adenosine monophosphate (cAMP) signaling, thereby promoting cyst growth and fluid secretion [2,3]. The clinical relevance of this pathway is supported by evidence that V2 receptor antagonism with tolvaptan slows kidney growth and decline in renal function in patients with ADPKD [4]. Because circulating AVP is unstable and technically challenging to measure, direct assessment of AVP activity is difficult. Copeptin, the C-terminal fragment of the AVP precursor peptide, is released in equimolar amounts with AVP and is substantially more stable in the circulation. It is therefore considered a useful surrogate biomarker of endogenous AVP secretion [5]. Circulating copeptin concentrations have been associated with renal outcomes in patients with type 2 diabetes and albuminuria, suggesting that copeptin may provide clinically relevant information on AVP system activity [6]. In ADPKD, the established role of the AVP–V2 receptor–cAMP pathway further supports the use of copeptin as a measurable marker of AVP activity [2,7]. Indeed, higher copeptin concentrations have been associated with kidney function decline and increases in total kidney volume in patients with ADPKD, supporting its potential value as a biomarker of disease progression [8,9].

Coffee is one of the most widely consumed beverages worldwide. Experimental studies have suggested that caffeine may increase intracellular cAMP accumulation in renal epithelial cells derived from patients with ADPKD, potentially promoting pathways involved in cyst epithelial cell proliferation and fluid secretion [3]. Studies in a *Pkd1*-deficient mouse model have suggested that caffeine consumption may accelerate cystic kidney disease, although renal cAMP levels did not differ between caffeine-treated and control animals [10]. However, clinical evidence regarding the effects of caffeine consumption on renal disease characteristics in ADPKD remains limited and inconsistent. For example, a study of 102 patients with ADPKD found no significant association between caffeine intake and renal volume [11]. The 2025 KDIGO Clinical Practice Guideline for ADPKD acknowledges that caffeine can accelerate cyst enlargement in experimental models, but caffeine restriction has not been shown to be effective in people with ADPKD. Accordingly, the guideline recommends limiting caffeine intake to less than 400 mg/day, consistent with recommendations for the general population [12]. More recently, an epidemiological and experimental study in the general population reported that higher coffee consumption was associated with lower plasma copeptin concentrations, while an experimental study in healthy volunteers showed a transient reduction in copeptin after consumption of 400 mL of coffee [13]. Whether a similar association exists in patients with ADPKD has not been established. Despite these findings, the relationship between habitual coffee consumption and serum copeptin, a clinically measurable surrogate marker of AVP activity, remains insufficiently studied in patients with ADPKD. Therefore, investigating the relationship between coffee consumption and serum copeptin may help clarify whether coffee intake is associated with a circulating marker of AVP system activity in patients with ADPKD.

Cardiovascular disease is a major cause of morbidity and mortality in patients with ADPKD [14]. Left ventricular hypertrophy (LVH) is among the most common cardiovascular manifestations and has been associated with hypertension and declining renal function [14,15]. Previous echocardiographic studies have demonstrated structural and functional cardiac abnormalities in patients with ADPKD, including increased left ventricular mass [15,16]. In addition to its established role as a marker of AVP activity, copeptin has also been investigated in relation to cardiovascular stress and cardiac function. In patients with heart failure and preserved ejection fraction, the KaRen study reported that copeptin was positively correlated with E/e′ and negatively correlated with e′ among patients with diastolic dysfunction [17]. Similarly, copeptin concentrations have been reported to be independently associated with E/e′ in patients receiving hemodialysis [18]. These findings suggest that copeptin may be related not only to renal and fluid-regulatory pathways but also to myocardial function and filling hemodynamics [17,18]. However, the relationship between copeptin and echocardiographic measures of diastolic function has not been sufficiently investigated in patients with ADPKD. Coffee consumption itself has also been investigated in relation to cardiac function. In the CARDIA study, low-to-moderate long-term coffee consumption was associated with a lower E/e′ ratio and some more favorable indices of cardiac function [19]. In contrast, in the Hamburg City Health Study including 9009 participants, different levels of coffee consumption were not significantly associated with any of the investigated echocardiographic parameters after adjustment for cardiovascular risk factors [20]. These inconsistent findings suggest that the relationship between coffee consumption and cardiac function may vary according to the population studied and the echocardiographic parameter evaluated [19,20]. In ADPKD, however, the potential relationships among coffee consumption, copeptin, and echocardiographic parameters of cardiac structure and function have not been adequately investigated.

Therefore, the primary objective of this study was to investigate the association between habitual coffee consumption and serum copeptin concentrations in patients with ADPKD. Secondary objectives were to examine the relationships between serum copeptin and echocardiographic parameters and to compare cardiac structural and functional parameters according to coffee consumption status. These secondary analyses were considered exploratory and were intended to generate hypotheses regarding the potential relationships among coffee consumption, vasopressin–copeptin system activity, and cardiac function in ADPKD, rather than to establish causal relationships.

## 2. Materials and Methods

**Figure 1:**
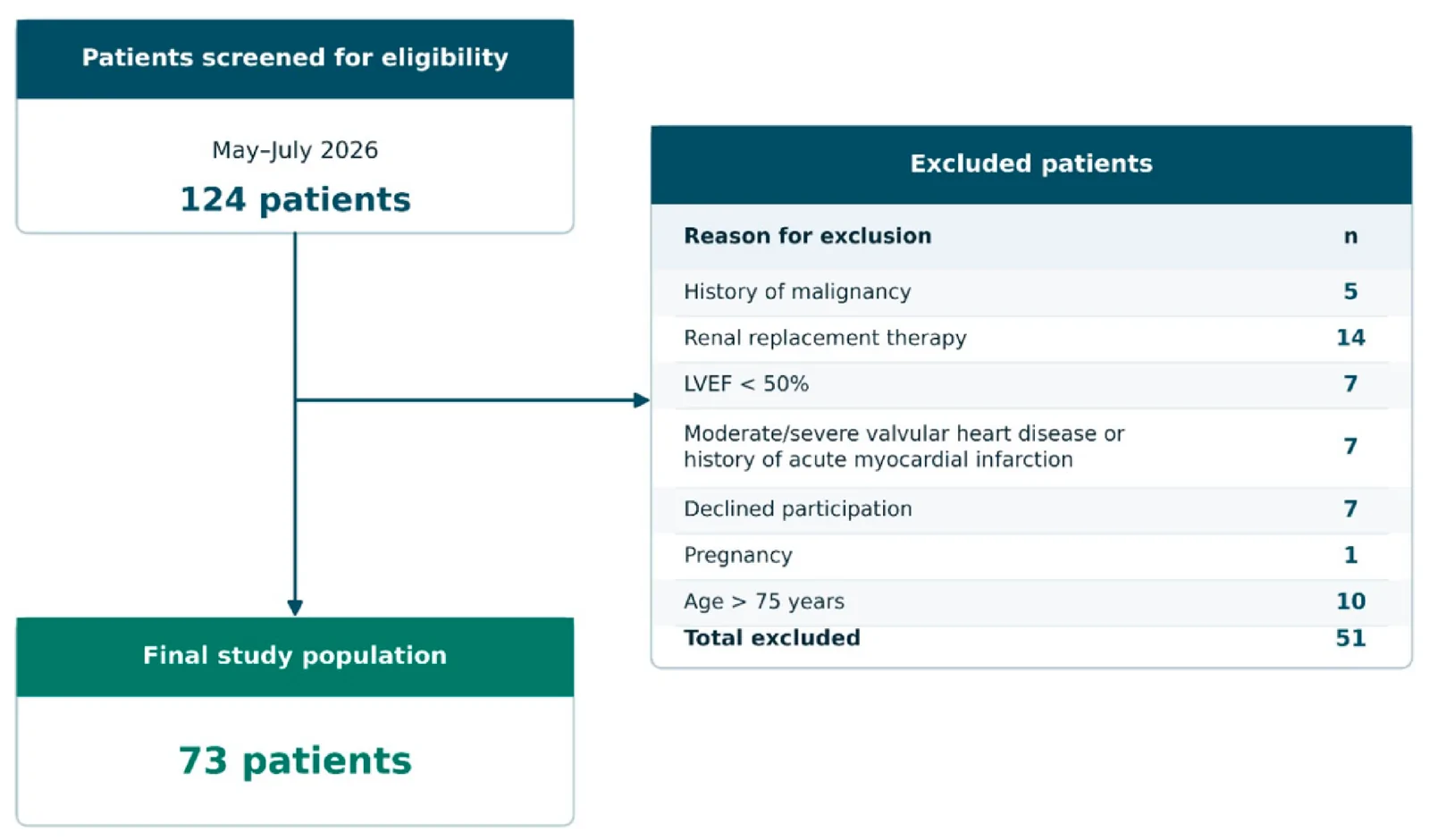
Flow diagram of patient selection and exclusion (LVEF: left ventricular ejection fraction).

From May to July 2026, a total of 124 patients with ADPKD registered in the Turkish Society of Nephrology Cystic Kidney Diseases Study Group database and evaluated at the Nephrology Outpatient Clinic of Bağcılar Training and Research Hospital were screened for eligibility. Five patients were excluded due to a history of malignancy, 14 because they had initiated renal replacement therapy, 7 because their left ventricular ejection fraction (LVEF) was <50%, 7 because of moderate or severe valvular heart disease or a history of acute myocardial infarction, 7 because they declined to participate in the study, 1 because of pregnancy, and 10 because they were older than 75 years. Thus, the final study population consisted of 73 patients. Serum copeptin measurements were available for 62 of the 73 patients included in the clinical and echocardiographic analyses. The patient selection process is summarized in Figure 1. Copeptin data were unavailable for 11 patients because of sample deterioration, refusal to provide an additional blood sample, or failure to return for repeat blood sampling.

Eligible participants were aged 18–75 years and were not receiving renal replacement therapy. The exclusion criteria were active infection, malignancy, rheumatologic disease, previous acute myocardial infarction, moderate or severe valvular heart disease, LVEF <50%, and pregnancy. All clinical, laboratory, coffee consumption, and echocardiographic assessments were performed during the same study visit, and the analyses were based on cross-sectional data.

The Medipol University Faculty of Medicine Non-Interventional Clinical Research Ethics Committee approved the study (Decision No. E-10840098-50.04-3293; 12 May 2026). The study was conducted in accordance with the principles of the Declaration of Helsinki, and written informed consent was obtained from all participants.

Venous blood samples were collected in the early morning, between 08:00 and 09:00, after at least 8 h of fasting, during which no food or fluids were consumed. Participants had not consumed coffee since the previous evening. Blood samples were centrifuged at 3000 revolutions per minute for 10 min. using a centrifuge (NF 400; Nüve, Ankara, Türkiye). Serum samples were stored at −80 °C in a deep freezer (DF 490; Nüve, Ankara, Türkiye) until analysis. Biochemical parameters were measured using an automated analyzer (Shenzhen Mindray Bio-Medical Electronics Co., Ltd., Shenzhen, China), hormonal parameters were analyzed using an ADVIA 1800 automated analyzer (Siemens Healthineers, Tarrytown, NY, USA), and hematological parameters were assessed using an XN-1000 hematology analyzer (Sysmex Corporation, Kobe, Japan). Serum copeptin concentrations were determined using a commercially available enzyme-linked immunosorbent assay (ELISA) kit (Human Copeptin ELISA, CEA365Hu; Cloud-Clone Corp., Katy, TX, USA) according to the manufacturer’s instructions. Spot urine samples were collected during the same visit, and the urinary albumin-to-creatinine ratio (UACR) was calculated. A UACR ≥30 mg/g was considered indicative of albuminuria [21].

Hypertension and diabetes mellitus were defined in accordance with current guidelines from the European Society of Cardiology (ESC) and the American Diabetes Association (ADA), respectively [22,23]. Blood pressure was recorded as the mean of two measurements obtained 15 min apart. Demographic characteristics, clinical information, smoking status, and concomitant medications were recorded during the study visit.

Coffee consumption was assessed using a standardized questionnaire administered during the study visit. Participants were asked to report the frequency and amount of Turkish coffee, filter coffee, and instant coffee consumed during the preceding month (1-month recall period) [24]. For the purposes of this study, participants who reported no coffee consumption during this period were classified as non-consumers, whereas participants who reported any coffee consumption during this period were classified as consumers. Reported intake was converted to standardized amounts of dry coffee and expressed as grams per month (g/month). Because coffee consumption expressed solely in cups may vary according to cup size and preparation method, we standardized reported intake according to the type of coffee consumed [25]. For the purposes of this study, predefined conversion factors of 7 g of dry coffee per cup of Turkish coffee, 10 g per serving of filter coffee, and 2 g per serving of instant coffee were applied. The amounts consumed for each coffee type were summed to calculate mean monthly coffee consumption. The resulting exposure variable was therefore expressed as standardized dry coffee intake (g/month) rather than estimated caffeine intake. As caffeine content varies according to coffee type and preparation method [26,27], the calculated coffee amount was not considered a direct measure of caffeine exposure. Accordingly, total monthly coffee consumption was used as the primary coffee exposure variable.

Transthoracic echocardiography was performed in accordance with the recommendations of the American Society of Echocardiography and the European Association of Cardiovascular Imaging by the same experienced cardiologist, who was blinded to the participants’ clinical and laboratory data [28]. Left ventricular end-diastolic diameter (LVDd), left ventricular end-systolic diameter (LVSd), left ventricular end-diastolic volume (LVEDV), left ventricular end-systolic volume (LVESV), left atrial diameter (LAD), posterior wall thickness (PWT), interventricular septal thickness (IVST), LVEF, fractional shortening (FS), aortic root diameter (Ao), ascending aortic diameter (AscAo), early (e′) and late (a′) diastolic mitral annular velocities, peak transmitral flow velocity (E), tricuspid annular plane systolic excursion (TAPSE), and systolic pulmonary artery pressure (sPAP) were assessed [28,29].

Left ventricular mass (LVM) was calculated using the Devereux formula and indexed to body surface area (BSA) to obtain LVMI. BSA was calculated using the Mosteller formula. Increased LVMI was defined according to the sex-specific upper reference limits recommended by the American Society of Echocardiography (>115 g/m^2^ in men and >95 g/m^2^ in women) [28]. Participants were subsequently classified into groups with normal or increased LVMI.

Estimated glomerular filtration rate (eGFR) was calculated using the 2021 Chronic Kidney Disease Epidemiology Collaboration (CKD-EPI) creatinine equation, which does not include a race coefficient [30].

### 2.1. Sample Size

No formal a priori sample size calculation was performed before the study. The sample size was determined by the number of eligible patients available during the predefined study period. As a post hoc sensitivity analysis, G*Power version 3.1.9.4 was used for the primary multivariable linear regression model of log-transformed serum copeptin levels. Based on 62 patients, six independent variables, a two-sided α of 0.05, and a target statistical power of 95%, the minimum detectable effect size for the additional contribution of coffee consumption was f^2^ = 0.217. This sensitivity analysis was not interpreted as evidence of 95% statistical power or as demonstrating adequate sample size for all analyses. The precision of the estimates was additionally assessed using 95% confidence intervals.

### 2.2. Statistical Analysis

Statistical analyses were performed using IBM SPSS Statistics for Windows, version 31.0 (IBM Corp., Armonk, NY, USA). Categorical variables were presented as numbers (*n*) and percentages (%), whereas continuous variables were presented as mean ± standard deviation and median (minimum–maximum). The normality of continuous variables was assessed using skewness and kurtosis coefficients. For the present sample size, absolute z-scores for skewness and kurtosis below 3.29 were considered compatible with an approximately normal distribution [31]. The independent-samples *t*-test was used to compare normally distributed continuous variables between the two groups, whereas the Mann–Whitney *U* test was used for non-normally distributed variables. Pearson’s chi-square test or Fisher’s exact test was used for categorical variables. Effect sizes were calculated and expressed as Cohen’s *d* for the independent-samples *t*-test and *r* = |*Z*|/√*N* for the Mann–Whitney *U* test.

Associations between coffee consumption status and clinical, laboratory, and echocardiographic variables were assessed using Spearman’s rank correlation analysis. Correlations involving coffee consumption status were evaluated in the entire study population, whereas correlations involving monthly coffee consumption were assessed only among coffee consumers. Because serum copeptin levels showed a skewed distribution, a natural logarithmic transformation was applied before multivariable linear regression analysis. Coffee consumption status was included as the primary independent variable in the multivariable linear regression model for log-transformed serum copeptin levels, together with age, sex, smoking status, total daily fluid intake, and eGFR. Potential confounders were selected based on their clinical relevance and their potential associations with coffee consumption, serum copeptin levels, renal function, and cardiovascular function. A sensitivity analysis was performed by replacing smoking status with pack-years. Among coffee consumers, the association between monthly coffee consumption and log-transformed serum copeptin levels was additionally evaluated using unadjusted and adjusted linear regression models.

Separate multivariable linear regression models were also constructed with mitral annular e′ velocity and the mitral E/e′ ratio as dependent variables. In both models, coffee consumption status was included as the primary independent variable, with age, sex, smoking status, hypertension, and eGFR included as covariates. All independent variables were entered simultaneously using the Enter method.

Missing data were excluded on an analysis-specific basis, and no imputation was performed. Missingness occurred at the variable level rather than as complete exclusion of participants; copeptin measurements were available for 62 patients and anthropometric measurements for 67 patients. Accordingly, regression analyses included only patients with complete data for all variables included in the respective model. Multicollinearity and model assumptions were assessed, and sensitivity analyses using HC3 heteroskedasticity-robust standard errors were performed. All statistical tests were two-sided, and *p* < 0.05 was considered statistically significant. Multiple secondary comparisons were considered exploratory, and unadjusted *p* values were reported.

## 3. Results

The demographic, clinical, lifestyle, and anthropometric characteristics of the study population according to coffee consumption status are presented in Table 1. A total of 73 patients with ADPKD were included, of whom 38 (52.1%) were non-coffee consumers and 35 (47.9%) were coffee consumers. The mean age of the study population was 47.85 ± 15.83 years. Overall, 41 patients (56.2%) were female, 32 patients (43.8%) were male, 13 (17.8%) had diabetes mellitus, 38 (52.1%) had hypertension, and 30 (41.1%) were smokers. No statistically significant differences were observed between the two groups for sex, age, diabetes mellitus, hypertension, disease duration, follow-up duration, total daily fluid intake, blood pressure, body mass index, height, body weight, total body water, or body surface area (all *p* > 0.05). Current smoking was more frequent among coffee consumers than non-coffee consumers (57.1% vs. 26.3%, *p* = 0.007), and smoking exposure was also higher in coffee consumers (18.62 ± 14.88 vs. 9.70 ± 8.68 pack-years, *p* = 0.045). Among coffee consumers, the mean monthly coffee consumption was 122.29 ± 133.55 g, with a median of 60 g/month (range, 10–540 g/month).

**Figure 2:**
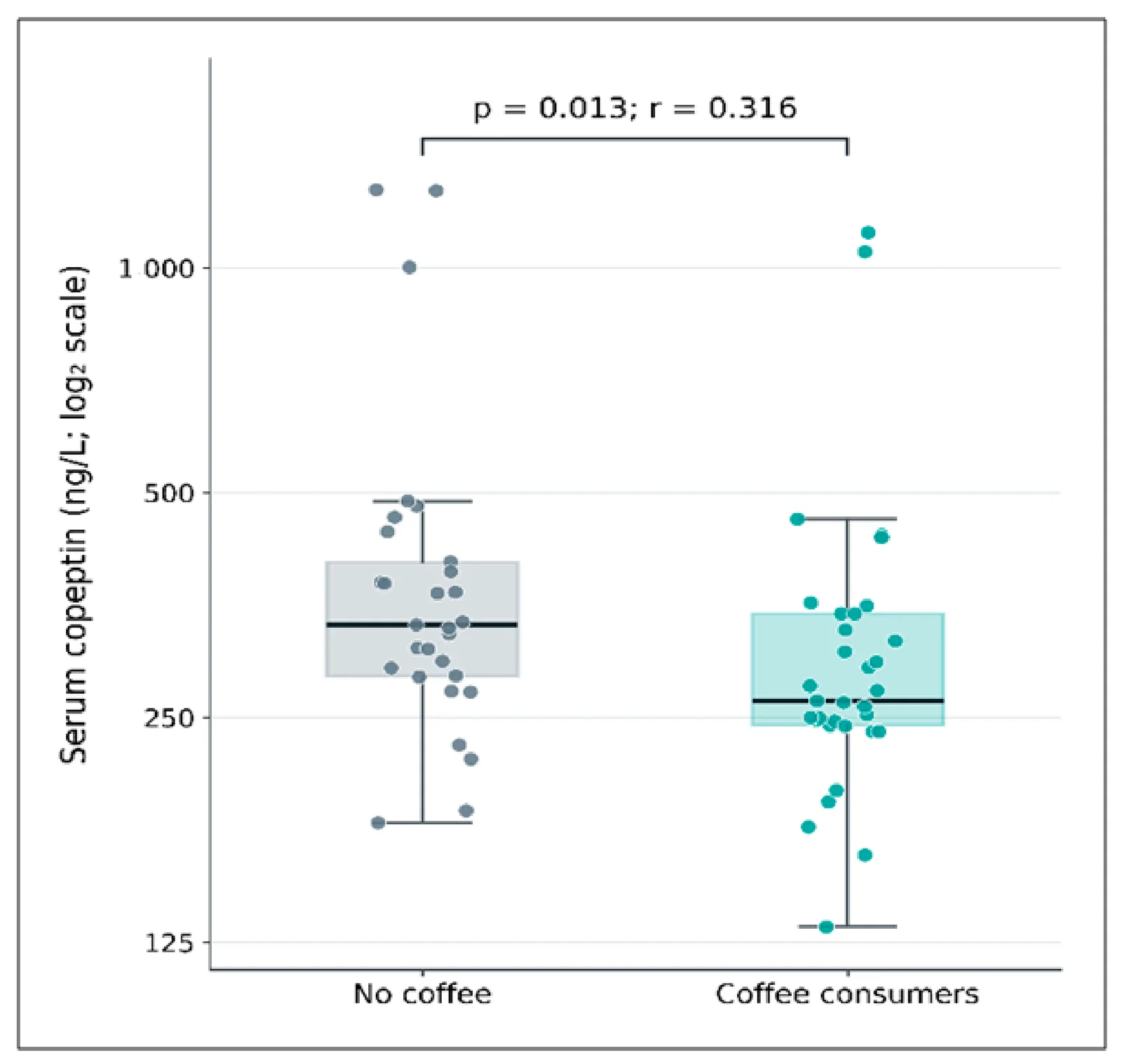
The distribution of serum copeptin levels.

The serum copeptin level and other laboratory parameters according to coffee consumption status are presented in Table 2. Serum copeptin levels were significantly lower among coffee consumers than among non-coffee consumers (median: 263.51 vs. 332.77 ng/L, *p* = 0.013; *r* = 0.316). As illustrated in Figure 2, copeptin levels were higher among non-coffee consumers. No significant differences were observed between the groups in glucose, urea, creatinine, eGFR, uric acid, calcium, sodium, potassium, phosphate, parathyroid hormone, leukocyte count, hemoglobin, hematocrit, platelet count, or spot urine albumin-to-creatinine ratio (all *p* > 0.05). Serum copeptin measurements were available for 62 of the 73 included patients; therefore, the comparison of copeptin levels was based on these 62 patients.

**Figure 3:**
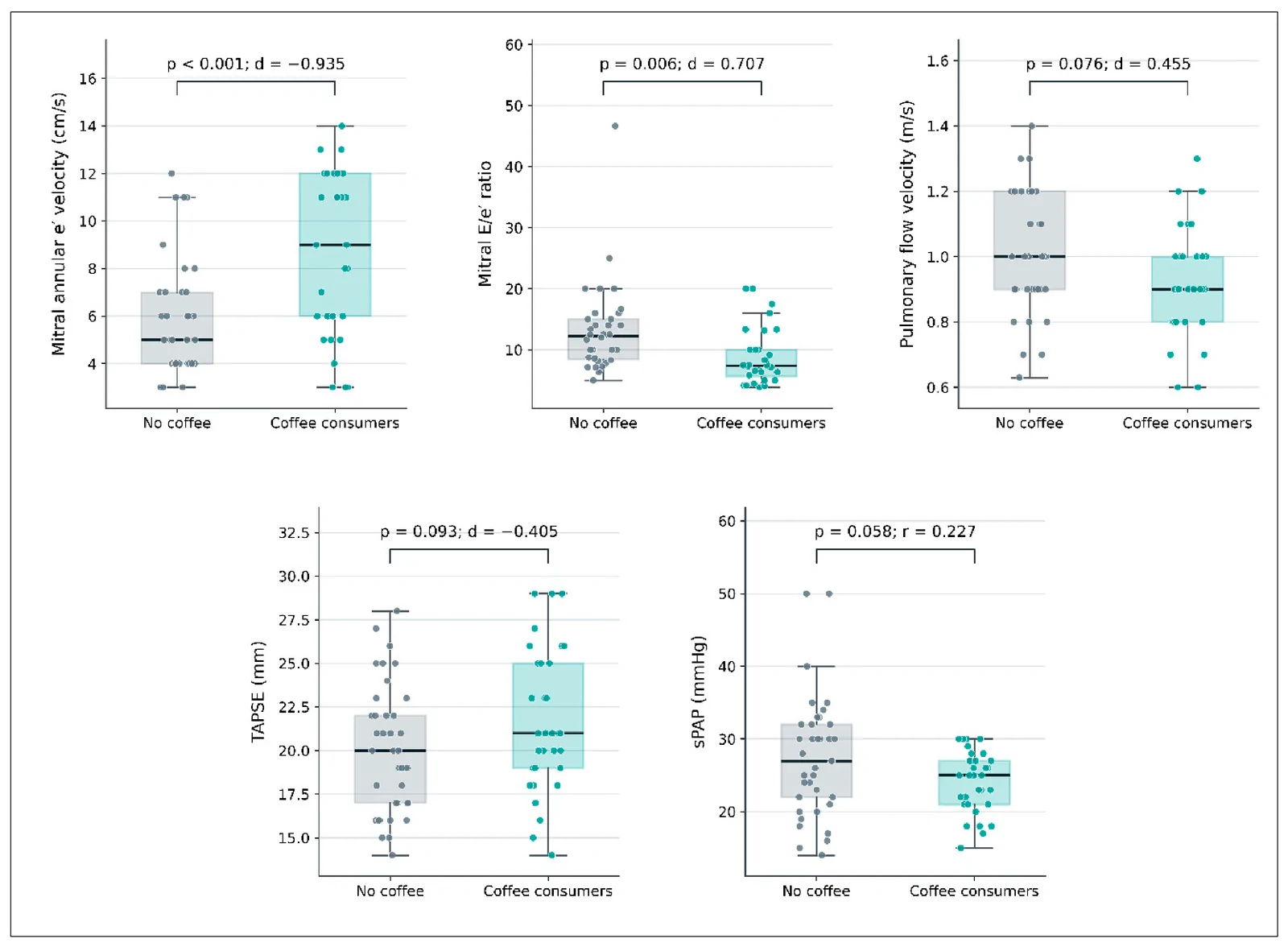
The distribution of selected cardiac functional and hemodynamic parameters according to coffee consumption status.

Echocardiographic parameters according to coffee consumption status are presented in Table 3. Mitral annular early diastolic tissue velocity (e′) was significantly higher in coffee consumers than in non-consumers (8.61 ± 3.43 vs. 5.86 ± 2.41 cm/s, *p* < 0.001; Cohen’s *d* = −0.935), indicating a large effect size. The mitral E/e′ ratio was significantly lower in coffee consumers than in non-consumers (8.75 ± 4.55 vs. 13.18 ± 7.52, *p* = 0.006; Cohen’s *d* = 0.707), indicating a moderate effect size. No statistically significant differences were observed between the groups in other cardiac structural measurements, left ventricular systolic or diastolic function parameters, valvular or flow parameters, systolic pulmonary artery pressure, left ventricular mass, or left ventricular mass index (all *p* > 0.05). The prevalence of mitral, aortic, and tricuspid regurgitation and increased left ventricular mass index also did not differ significantly between the groups (all *p* > 0.05). Selected cardiac functional and hemodynamic parameters are illustrated in Figure 3.

Correlation analyses were performed to further evaluate the relationships between coffee consumption and selected clinical, biochemical, and echocardiographic variables (Table 4). Coffee consumption status was positively correlated with smoking status (*r* = 0.313, *p* = 0.007) and mitral annular early diastolic tissue velocity (e′) (*r* = 0.408, *p* < 0.001), whereas inverse correlations were observed with serum copeptin levels (*r* = −0.319, *p* = 0.012) and the E/e′ ratio (*r* = −0.414, *p* < 0.001).

Among coffee consumers, monthly coffee consumption was positively correlated with smoking status (*r* = 0.380, *p* = 0.025), mitral regurgitation (*r* = 0.549, *p* < 0.001), age (*r* = 0.442, *p* = 0.008), follow-up duration (*r* = 0.423, *p* = 0.011), and serum copeptin levels (*r* = 0.393, *p* = 0.024) and mitral late diastolic flow velocity (A) (*r* = 0.352, *p* = 0.044). Monthly coffee consumption amount was not significantly correlated with e′ (*r* = 0.044, *p* = 0.807) or the E/e′ ratio (*r* = −0.058, *p* = 0.752).

Given the skewed distribution of serum copeptin levels, a natural logarithmic transformation was applied before regression analysis. In the correlation analysis, coffee consumption status was inversely correlated with serum copeptin levels (Spearman’s *r* = −0.319, *p* = 0.012), whereas, among coffee consumers, monthly coffee consumption was positively correlated with serum copeptin levels (*r* = 0.393, *p* = 0.024) (Table 4). These findings were considered separately because coffee consumption status and monthly coffee consumption amount represent different exposure measures.

To further evaluate the association observed between monthly coffee consumption and serum copeptin levels among coffee consumers, additional regression analyses were performed. The results of the unadjusted model and the model adjusted for age, smoking status, and eGFR are presented in Table 5. Age and smoking status were selected as adjustment variables because they were associated with coffee consumption amount, whereas eGFR was included as a marker of kidney function.

Among 33 coffee-consuming patients with complete data, the unadjusted model showed a significant positive association between LN (coffee consumption amount) and LN(copeptin) (B = 0.154; β = 0.408; 95% CI: 0.028–0.280; *p* = 0.018; R^2^ = 0.167). After adjustment for age, smoking status, and eGFR, the coefficient for coffee consumption amount remained positive but was no longer statistically significant (B = 0.133; β = 0.353; 95% CI: −0.018–0.284; *p* = 0.082). The adjusted model was also not statistically significant overall [F(4,28) = 2.180; *p* = 0.097].

Therefore, the positive association observed between coffee consumption amount and copeptin levels among coffee consumers was not confirmed after adjustment. However, the persistence of a positive coefficient and the wide confidence interval do not allow us to conclude that the association was absent or entirely attributable to confounding. Given the small sample size and cross-sectional design, these findings were interpreted cautiously.

A multivariable linear regression model was constructed to examine the association between coffee consumption status and ln(copeptin), adjusting for age, sex, smoking status, total daily fluid intake, and eGFR (Table 6). The model explained 14.9% of the variance in ln(copeptin) (R^2^ = 0.149; adjusted R^2^ = 0.056), although the overall model was not statistically significant [F(6,55) = 1.606, *p* = 0.163]. In the conventional regression analysis, coffee consumption was associated with lower ln(copeptin) levels (B = −0.263, β = −0.286, 95% CI: −0.522 to −0.004; *p* = 0.047), corresponding to an approximately 23.1% lower adjusted geometric mean copeptin level among coffee consumers (ratio = 0.769, 95% CI: 0.593–0.997). However, this association did not remain statistically significant when HC3 robust standard errors were applied (*p* = 0.074), and the finding was therefore interpreted cautiously.

A sensitivity analysis was performed by replacing smoking status with cumulative smoking exposure, expressed as pack-years, while retaining age, sex, total daily fluid intake, and eGFR in the model (Table 7). The model explained 14.6% of the variance in ln(copeptin) (R^2^ = 0.146; adjusted R^2^ = 0.053) and was not statistically significant overall [F(6,55) = 1.571, *p* = 0.173]. In the sensitivity analysis, the association between coffee consumption and ln(copeptin) remained negative but was no longer statistically significant (B = −0.248, β = −0.270, 95% CI: −0.510 to 0.015; *p* = 0.064). Smoking exposure and the other covariates were not significantly associated with ln(copeptin) (all *p* > 0.05). VIF values ranged from 1.141 to 2.065, indicating no substantial multicollinearity.

Compared with the main model using smoking status, the coefficient for coffee consumption remained similar in direction and magnitude, although statistical significance was not maintained. This finding indicates that the statistical significance of the association between coffee consumption and ln(copeptin) was sensitive to how smoking exposure was modeled. However, the result was not interpreted as evidence that the association was entirely attributable to smoking exposure. The assignment of 0 pack-years to participants who reported no smoking but had missing pack-year records was considered an assumption of this sensitivity analysis.

Two multivariable linear regression models were constructed to evaluate the associations of coffee consumption with mitral annular e′ velocity and the mitral E/e′ ratio, with each parameter analyzed separately as the dependent variable (Table 8). Both models were statistically significant overall. The e′ velocity model explained 29.5% of the variance (R^2^ = 0.295; adjusted R^2^ = 0.227; *p* < 0.001), whereas the E/e′ ratio model explained 21.6% of the variance (R^2^ = 0.216; adjusted R^2^ = 0.136; *p* = 0.022).

After adjustment for age, sex, smoking status, hypertension, and eGFR, coffee consumption was associated with higher mitral annular e′ velocity (B = 2.531, β = 0.395, 95% CI: 1.081–3.981; *p* < 0.001). Age was also inversely associated with e′ velocity (B = −0.066; *p* = 0.021), whereas the other covariates were not significantly associated with e′ velocity (all *p* > 0.05).

In the E/e′ ratio model, coffee consumption was associated with a lower E/e′ ratio after adjustment (B = −4.548, β = −0.347, 95% CI: −7.786 to −1.310; *p* = 0.007). Hypertension was also associated with a lower E/e′ ratio (B = −3.404, β = −0.260, 95% CI: −6.655 to −0.153; *p* = 0.040), whereas age, sex, smoking status, and eGFR were not significantly associated with E/e′ (all *p* > 0.05). VIF values did not indicate substantial multicollinearity in either model.

## 4. Discussion

In this study, we evaluated the relationships between coffee consumption, serum copeptin levels, and cardiac parameters in patients with autosomal dominant polycystic kidney disease (ADPKD). Coffee consumers had significantly lower serum copeptin levels and higher mitral annular early diastolic tissue velocity (e′) than non-consumers. The association between coffee consumption and e′ remained statistically significant after multivariable adjustment, and coffee consumption was also associated with a lower E/e′ ratio following multivariable adjustment, whereas no significant associations were observed with left ventricular mass index (LVMI), left ventricular ejection fraction (LVEF), or other major echocardiographic parameters. Coffee consumption was inversely correlated with serum copeptin levels, whereas monthly coffee consumption was positively correlated with copeptin among coffee consumers. The echocardiographic findings were exploratory, and the observed associations should be interpreted in the context of the cross-sectional study design.

Experimental studies have suggested that caffeine may accelerate cyst growth in ADPKD, providing a rationale for recommendations to limit caffeine intake [3,10]. However, clinical evidence regarding coffee and caffeine consumption remains inconsistent [11,32,33]. Accordingly, the 2025 KDIGO ADPKD guideline recommends limiting caffeine intake to less than 400 mg/day, while acknowledging that caffeine restriction has not been shown to be effective in people with ADPKD [12].

In ADPKD, higher circulating copeptin levels have been associated with greater kidney volume and subsequent kidney growth [9,34]. In the CRISP cohort, baseline copeptin was independently associated with change in total kidney volume during long-term follow-up [9]. Similarly, in the TEMPO 3:4 trial, higher baseline copeptin predicted kidney growth and eGFR decline, although these associations were attenuated after adjustment for baseline total kidney volume [34]. These findings support copeptin as a marker of disease severity and progression, consistent with the proposed role of vasopressin signaling in ADPKD [7,8,9,34].

Echocardiographic studies in ADPKD have mainly demonstrated left ventricular hypertrophy and diastolic dysfunction, which may occur even in patients with preserved renal function [14,15,16]. Increased LV mass, impaired relaxation, left atrial enlargement, and other abnormalities of diastolic function have been reported, although findings across studies are not entirely consistent [14,15,16]. Overall, the available evidence suggests that cardiac involvement, particularly abnormalities of diastolic function, may be part of the cardiovascular phenotype of ADPKD [14,15,16].

Taken together, these observations provided the rationale for our hypothesis that coffee consumption might be associated with vasopressin-related activity, reflected by serum copeptin levels, and with cardiac function, particularly indices of diastolic function, in patients with ADPKD.

In our study, serum copeptin levels were significantly lower in coffee-consuming patients with ADPKD than in non-consumers, suggesting a possible relationship between coffee consumption and the vasopressin system. This finding is consistent with the results reported by Schill et al. [13] in an epidemiological analysis of 3270 participants from the Malmö Offspring Study, in which higher coffee consumption was associated with lower plasma copeptin concentrations after adjustment for total fluid intake and other potential confounding factors. In the experimental component of the same study, copeptin concentrations decreased significantly after consumption of 400 mL of coffee, with a maximum reduction of approximately 27% at 150 min [13]. In the present study, blood samples were collected in the early morning after at least 8 h of fasting, and the participants had not consumed coffee since the previous evening. Therefore, the observed differences in copeptin levels were assessed under standardized fasting and pre-sampling conditions, although no standardized hydration protocol was applied, and were primarily intended to reflect habitual coffee consumption rather than acute coffee intake.

Notably, water consumption also produced a more modest reduction in copeptin concentrations, suggesting that the observed effect may not be attributable exclusively to caffeine [13]. In our study, caffeine intake was not directly quantified; therefore, the lower copeptin concentrations could not be specifically attributed to caffeine, other coffee components, or differences in fluid intake. Importantly, the positive correlation between monthly coffee consumption and serum copeptin levels among coffee consumers (*r* = 0.393, *p* = 0.024) does not support a simple linear dose–response relationship. Coffee consumption status and coffee amount among consumers represent different exposure comparisons and therefore should not be interpreted as equivalent measures of dose–response. In the additional regression analysis, the positive association between log-transformed monthly coffee consumption and log-transformed copeptin was no longer statistically significant after adjustment for age, smoking status, and eGFR (B = 0.133, *p* = 0.082). This finding should be interpreted cautiously given the small number of coffee consumers (*n* = 35) and the observed differences in smoking exposure. Moreover, the higher prevalence and cumulative exposure to smoking among coffee consumers may represent an additional source of residual confounding, despite adjustment for smoking in the multivariable model. In the sensitivity analysis using pack-years instead of smoking status, the association between coffee consumption and log-transformed copeptin was attenuated and did not reach statistical significance (*p* = 0.064), further supporting cautious interpretation of this finding. Residual confounding and differences in coffee type and preparation may also have contributed to this finding.

The potential importance of copeptin in ADPKD is closely related to the established role of the vasopressin system in disease progression. Copeptin is released in equimolar amounts with arginine vasopressin (AVP) and, because of its greater stability in the circulation, is widely used as a surrogate marker of AVP secretion [5]. AVP activates the V2 receptor and increases intracellular cAMP signaling, thereby promoting cyst growth and fluid secretion in ADPKD [35]. Pharmacological inhibition of this pathway with the V2-receptor antagonist tolvaptan has been shown to slow kidney enlargement and loss of renal function [4]. Because total kidney volume and longitudinal renal outcomes were not assessed in the present study, the clinical significance of the observed copeptin difference remains uncertain. Previous studies of coffee and copeptin have primarily involved non-ADPKD populations; therefore, whether this association is specific to ADPKD cannot be determined from our findings.

Clinical evidence regarding the effects of coffee and caffeine consumption on renal outcomes in ADPKD has not demonstrated a consistent adverse effect on kidney function. Vendramini et al. found no significant association between caffeine consumption and renal volume or eGFR [11]. Similarly, Girardat-Rotar et al. reported no significant association between coffee consumption and height-adjusted total kidney volume (htTKV) or eGFR in a prospective longitudinal ADPKD cohort [32]. In a longer-term analysis of the CRISP cohort, caffeine consumption was associated with an approximately 0.6% annual increase in htTKV growth, but no significant associations were observed with measured GFR, end-stage kidney disease, or mortality [33]. Consistent with these findings, we observed no significant association between coffee consumption and eGFR. However, the small sample size and cross-sectional design preclude conclusions regarding the long-term renal effects of coffee or caffeine consumption.

In our study, coffee-consuming patients with ADPKD had higher e′ velocities than non-consumers, and coffee consumption was associated with both higher e′ velocities and lower E/e′ ratios after multivariable adjustment, suggesting a possible association between coffee consumption and selected indices of left ventricular diastolic function. In a study evaluating the acute effects of 100 mg of caffeine in healthy individuals, caffeine alone did not significantly alter ventricular long-axis function, whereas smoking was associated with acute changes in ventricular diastolic function [36]. In contrast, the CARDIA study reported that low-to-moderate coffee consumption was associated with lower E/e′ and more favorable global longitudinal strain, whereas total caffeine intake was not significantly associated with cardiac function [19]. Similarly, in a large population-based study of 9009 participants from the Hamburg City Health Study, coffee consumption was not significantly associated with the investigated echocardiographic parameters after adjustment for cardiovascular risk factors [20]. A population-based study using cardiac magnetic resonance imaging also reported associations between habitual coffee consumption and selected cardiac functional parameters, although the imaging modality and outcomes differed from those evaluated in the present study [37]. These differing findings may partly reflect variations in study populations, the dose and assessment of coffee or caffeine exposure, smoking and other cardiovascular risk factors, and the specific echocardiographic parameters evaluated, such as e′ and E/e′. The relationship between habitual coffee consumption and these echocardiographic parameters has not previously been directly examined in this population. Our study therefore provides preliminary data regarding this association within an ADPKD cohort. The underlying mechanism remains uncertain, and because caffeine intake was not directly quantified, the observed association cannot be specifically attributed to caffeine.

Coffee consumption was associated with higher e′ and lower E/e′, but was not significantly associated with LVMI, LVEF, or other major echocardiographic parameters, suggesting that the observed associations were limited to selected diastolic indices rather than representing a consistent change across cardiac parameters. Therefore, the higher e′ velocity and lower E/e′ ratio should not be interpreted as evidence of a direct cardioprotective effect of coffee. Prospective studies with standardized assessment of coffee and caffeine intake and serial echocardiographic measurements are needed to determine whether these findings are reproducible and clinically meaningful.

Several limitations should be considered when interpreting our findings. First, the cross-sectional design precludes establishing temporal or causal relationships. Second, caffeine intake was not directly quantified, and differences in caffeine content related to coffee type and preparation could not be fully accounted for. Third, total kidney volume and longitudinal measures of ADPKD progression were not available, limiting assessment of the long-term clinical significance of the observed copeptin findings. Fourth, the relatively small sample size, particularly the limited number of coffee consumers in the correlation analyses, may have reduced statistical power and increased the uncertainty of some estimates. Fifth, coffee consumption was self-reported and assessed at a single time point, and recall bias cannot be excluded. Finally, residual confounding, particularly from smoking and other lifestyle factors, cannot be completely excluded, despite adjustment for selected covariates. The single-center design may also limit generalizability.

## 5. Conclusions

In this cross-sectional study of patients with ADPKD, coffee consumption was associated with lower serum copeptin levels and selected echocardiographic parameters, including higher mitral annular e′ velocity and lower E/e′ ratio. No significant association was observed with eGFR or other major echocardiographic parameters. These findings provide preliminary evidence of an association between coffee consumption, serum copeptin levels, and selected indices of diastolic function in patients with ADPKD; however, because these associations have not been similarly evaluated in healthy individuals, whether the observed findings are specific to ADPKD cannot be determined. These findings should be considered hypothesis-generating and require confirmation in larger, prospective studies.

## Figures and Tables

**Table 1:** Comparison of demographic, clinical, lifestyle, and anthropometric characteristics of patients with autosomal dominant polycystic kidney disease according to coffee consumption status.

Variable	Group	Total (*n* = 73)	Non- Consumers (*n* = 38)	Coffee Consumers (*n* = 35)	*p*
Sex	Male	32 (43.8)	16 (42.1)	16 (45.7)	0.756
	Female	41 (56.2)	22 (57.9)	19 (54.3)	
Diabetes mellitus	No	60 (82.2)	29 (76.3)	31 (88.6)	0.172
	Yes	13 (17.8)	9 (23.7)	4 (11.4)	
Hypertension	No	35 (47.9)	16 (42.1)	19 (54.3)	0.298
	Yes	38 (52.1)	22 (57.9)	16 (45.7)	
Smoking status	No	43 (58.9)	28 (73.7)	15 (42.9)	**0.007** *
	Yes	30 (41.1)	10 (26.3)	20 (57.1)	
		Mean ± SD Median (Min–Max)	Mean ± SD Median (Min–Max)	Mean ± SD Median (Min–Max)	*p*
Age (years) ^t^	—	47.85 ± 15.83 46.00 (18.00–75.00)	49.16 ± 17.66 45.00 (18.00–75.00)	46.43 ± 13.68 46.00 (24.00–72.00)	0.461
Disease duration (months) ^z^	—	66.51 ± 75.38 36.00 (1.00–360.00)	74.26 ± 89.19 38.00 (1.00–360.00)	58.09 ± 56.90 36.00 (1.00–240.00)	0.947
Follow-up duration (months) ^t^	—	35.90 ± 35.67 24.00 (1.00–144.00)	34.79 ± 38.68 14.00 (1.00–144.00)	37.11 ± 32.61 24.00 (1.00–120.00)	0.783
Monthly coffee consumption among consumers (g/month) ^z^	—	—	—	122.29 ± 133.55 60.00 (10.00–540.00)	—
Smoking exposure (pack-years) ^t^	—	15.74 ± 13.72 15.00 (1.00–55.00)	9.70 ± 8.68 7.50 (1.00–25.00)	18.62 ± 14.88 20.00 (1.00–55.00)	**0.045** *
Total daily fluid intake (L/day) ^t^	—	2.36 ± 0.65 2.00 (2.00–4.00)	2.32 ± 0.66 2.00 (2.00–4.00)	2.40 ± 0.65 2.00 (2.00–4.00)	0.586
Systolic blood pressure (mmHg) ^t^	—	127.26 ± 14.77 125.00 (90.00–160.00)	128.68 ± 14.69 125.00 (100.00–160.00)	125.71 ± 14.91 125.00 (90.00–150.00)	0.394
Diastolic blood pressure (mmHg) ^t^	—	76.89 ± 8.75 80.00 (60.00–100.00)	77.24 ± 9.13 80.00 (60.00–100.00)	76.51 ± 8.43 80.00 (60.00–100.00)	0.727
Body mass index (kg/m^2^) ^t^	—	28.29 ± 4.77 29.00 (17.90–44.40)	27.99 ± 4.42 28.65 (17.90–35.40)	28.59 ± 5.15 29.10 (20.00–44.40)	0.612
Height (cm) ^t^	—	164.04 ± 8.60 163.00 (147.00–187.00)	162.41 ± 8.31 161.00 (147.00–177.00)	165.73 ± 8.70 163.00 (150.00–187.00)	0.115
Body weight (kg) ^t^	—	76.20 ± 13.57 76.80 (50.00–109.00)	73.84 ± 11.59 73.05 (52.40–99.90)	78.64 ± 15.14 79.80 (50.00–109.00)	0.149
Total body water (kg) ^t^	—	40.11 ± 7.37 38.10 (29.40–58.20)	38.76 ± 6.52 37.10 (29.90–53.10)	41.49 ± 8.02 40.80 (29.40–58.20)	0.131
Total body water (%) ^t^	—	53.04 ± 7.39 53.10 (40.31–70.50)	52.92 ± 7.61 52.66 (41.50–67.30)	53.17 ± 7.26 54.40 (40.31–70.50)	0.888
Body surface area (m^2^) ^t^	—	1.86 ± 0.19 1.85 (1.47–2.32)	1.82 ± 0.16 1.81 (1.53–2.16)	1.90 ± 0.21 1.90 (1.47–2.32)	0.109

Note: Data are presented as number (*n*) and percentage (%) for categorical variables and as mean ± standard deviation and median (minimum–maximum) for continuous variables. Pearson’s chi-square test or Fisher’s exact test was used for comparisons of categorical variables. ^t^: Independent-samples *t*-test; Welch’s *t*-test was applied for age and smoking exposure. ^z^: Mann–Whitney *U* test. Coffee consumption amount was assessed only among the 35 coffee consumers. Anthropometric measurements were available for 67 patients (34 non-coffee consumers and 33 coffee consumers). Missing data were excluded from the respective analyses. SD: standard deviation. Bold *p* values indicate statistically significant differences (* *p* < 0.05).

**Table 2:** Serum Copeptin Levels and Laboratory Parameters According to Coffee Consumption Status in Patients with Autosomal Dominant Polycystic Kidney Disease.

Variable	No Coffee Consumption (*n* = 38) Mean ± SD; Median (Min–Max)	Coffee Consumption (*n* = 35) Mean ± SD; Median (Min–Max)	*p*-Value	Effect Size
Serum copeptin level (ng/L) ^z^	419.45 ± 277.97 332.77 (180.85–1272.41)	328.83 ± 208.51 263.51 (131.19–1114.91)	**0.013** *	*r* = 0.316
Glucose (mg/dL) ^z^	117.64 ± 94.29 95.45 (82.50–660.00)	99.53 ± 16.61 97.20 (78.60–162.00)	0.938	*r* = 0.009
Urea (mg/dL) ^t^	41.14 ± 22.27 33.05 (15.10–108.50)	41.73 ± 20.46 33.00 (18.50–107.00)	0.907	*d* = −0.028
Creatinine (mg/dL) ^z^	1.07 ± 0.58 0.90 (0.46–2.85)	1.15 ± 0.55 1.00 (0.60–3.00)	0.339	*r* = 0.112
eGFR (mL/min/1.73 m^2^) ^t^	85.20 ± 32.53 87.33 (19.29–128.70)	81.13 ± 31.09 79.66 (16.02–127.45)	0.587	*d* = 0.128
Uric acid (mg/dL) ^t^	5.36 ± 1.93 5.35 (1.90–10.20)	5.40 ± 1.68 5.50 (2.40–11.50)	0.932	*d* = −0.020
Calcium (mg/dL) ^t^	9.60 ± 0.48 9.56 (8.40–10.90)	9.49 ± 0.39 9.47 (8.40–10.10)	0.302	*d* = 0.244
Sodium (mmol/L) ^t^	141.92 ± 3.01 142.50 (131.00–149.00)	141.17 ± 3.05 142.00 (135.00–146.00)	0.295	*d* = 0.247
Potassium (mmol/L) ^t^	4.51 ± 0.34 4.50 (3.69–5.10)	4.44 ± 0.30 4.40 (3.93–5.00)	0.390	*d* = 0.203
Phosphate (mg/dL) ^t^	3.31 ± 0.56 3.30 (2.20–4.40)	3.20 ± 0.56 3.26 (2.00–4.79)	0.409	*d* = 0.195
Parathyroid hormone (pg/mL) ^z^	80.70 ± 63.12 55.90 (26.40–256.00)	79.31 ± 51.31 65.10 (21.00–208.00)	0.659	*r* = 0.052
Leukocyte count (×10^3^/µL) ^t^	7.01 ± 1.76 6.80 (3.53–11.40)	7.13 ± 1.71 7.07 (4.28–11.80)	0.772	*d* = −0.069
Hemoglobin (g/dL) ^t^	13.44 ± 1.55 13.10 (9.70–16.40)	13.52 ± 1.64 13.30 (10.20–17.30)	0.826	*d* = −0.052
Hematocrit (%) ^t^	40.84 ± 4.74 40.40 (28.90–49.40)	41.83 ± 4.61 40.70 (33.80–53.50)	0.372	*d* = −0.212
Platelet count (×10^3^/µL) ^t^	257.71 ± 57.90 248.00 (173.90–412.00)	233.30 ± 57.38 238.00 (111.40–372.00)	0.077	*d* = 0.423
Spot urine albumin-to- creatinine ratio (mg/g) ^z^	77.47 ± 130.58 17.25 (2.50–634.30)	62.62 ± 116.29 21.80 (2.50–578.60)	0.647	*r* = 0.054

Note: Data are presented as mean ± standard deviation and median (minimum–maximum). Normally distributed continuous variables were compared using the independent-samples *t*-test, and non-normally distributed continuous variables were compared using the Mann–Whitney *U* test. Effect sizes were expressed as Cohen’s *d* for variables analyzed using the independent-samples *t*-test and as *r* = |Z|/√N for variables analyzed using the Mann–Whitney *U* test. For Cohen’s *d*, values of 0.20, 0.50, and 0.80 represent small, moderate, and large effects, respectively; for *r*, values of 0.10, 0.30, and 0.50 represent small, moderate, and large effects, respectively. ^t^ Independent-samples *t*-test; ^z^ Mann–Whitney *U* test; eGFR, estimated glomerular filtration rate. Serum copeptin measurements were available for 62 of the 73 included patients; copeptin data were unavailable for 11 patients. Bold values indicate statistically significant differences (* *p* < 0.05).

**Table 3:** Comparison of Echocardiographic Parameters According to Coffee Consumption Status in Patients with Autosomal Dominant Polycystic Kidney Disease.

Variable	No Coffee Consumption (*n* = 38) Mean ± SD; Median (Min–Max)	Coffee Consumption (*n* = 35) Mean ± SD; Median (Min–Max)	*p*-Value	Effect Size
Mitral regurgitation, *n* (%)				—
No	21 (55.3)	18 (51.4)	0.743	
Yes	17 (44.7)	17 (48.6)		
Aortic regurgitation, *n* (%)				—
No	28 (73.7)	26 (74.3)	0.953	
Yes	10 (26.3)	9 (25.7)		
Tricuspid regurgitation, *n* (%)				—
No	3 (7.9)	2 (5.7)	1.000	
Yes	35 (92.1)	33 (94.3)		
Increased left ventricular mass index (LVMI), *n* (%)				—
Normal/Not increased	18 (47.4)	22 (62.9)	0.184	
Increased	20 (52.6)	13 (37.1)		
Left ventricular end-diastolic diameter (LVDd), cm ^z^	4.72 ± 0.47; 4.65 (3.60–5.50)	4.81 ± 0.40; 4.80 (4.20–5.80)	0.535	*r* = 0.073
Left ventricular end-systolic diameter (LVSd), cm ^z^	3.11 ± 0.46; 3.05 (2.10–4.00)	3.15 ± 0.41; 3.10 (2.40–4.30)	0.916	*r* = 0.012
Interventricular septal thickness (IVST), cm ^t^	1.19 ± 0.27; 1.12 (0.80–1.90)	1.11 ± 0.19; 1.10 (0.70–1.55)	0.172	*d* = 0.323
Left ventricular posterior wall thickness (PWT), cm ^t^	1.06 ± 0.21; 1.00 (0.70–1.70)	1.06 ± 0.19; 1.00 (0.60–1.44)	0.971	*d* = −0.008
Left ventricular ejection fraction (LVEF), % ^z^	62.76 ± 4.64; 61.50 (51.00–72.00)	62.94 ± 5.54; 61.00 (52.00–75.00)	0.978	*r* = 0.003
Fractional shortening (FS), % ^z^	34.58 ± 4.04; 34.50 (26.00–48.00)	34.54 ± 4.42; 33.00 (27.00–45.00)	0.760	*r* = 0.036
Left ventricular end-diastolic volume (LVEDV), mL ^z^	115.00 ± 21.45; 121.00 (55.00–152.00)	115.97 ± 18.39; 117.00 (77.00–147.00)	0.922	*r* = 0.012
Left ventricular end-systolic volume (LVESV), mL ^z^	44.62 ± 10.65; 45.00 (21.00–65.00)	42.56 ± 11.20; 43.00 (25.00–67.00)	0.447	*r* = 0.090
Aortic root diameter, cm ^t^	2.69 ± 0.31; 2.65 (2.10–3.40)	2.67 ± 0.29; 2.70 (2.00–3.50)	0.715	*d* = 0.086
Ascending aortic diameter (AscAo), cm ^t^	3.23 ± 0.50; 3.30 (2.40–4.30)	3.22 ± 0.45; 3.20 (2.40–4.50)	0.934	*d* = 0.024
Left atrial diameter (LAD), cm ^z^	3.37 ± 0.77; 3.30 (0.60–5.70)	3.40 ± 0.48; 3.30 (2.60–4.80)	0.987	*r* = 0.002
Mitral early diastolic flow velocity (E), cm/s ^z^	68.00 ± 21.00; 70.00 (40.00–140.00)	64.00 ± 16.00; 60.00 (40.00–110.00)	0.474	*r* = 0.087
Mitral late diastolic flow velocity (A), cm/s ^t^	69.00 ± 14.00; 70.00 (40.00–110.00)	66.00 ± 13.00; 70.00 (40.00–90.00)	0.311	*d* = 0.243
Mitral annular early diastolic tissue velocity (e′), cm/s ^t^	5.86 ± 2.41; 5.00 (3.00–12.00)	8.61 ± 3.43; 9.00 (3.00–14.00)	**<0.001** *	*d* = −0.935
Mitral E/e′ ratio ^t^	13.18 ± 7.52; 12.25 (5.00–46.67)	8.75 ± 4.55; 7.39 (3.85–20.00)	**0.006** *	*d* = 0.707
Aortic flow velocity, m/s ^t^	1.09 ± 0.42; 1.10 (0.40–1.80)	1.25 ± 0.25; 1.20 (0.60–1.70)	0.111	*d* = −0.444
Pulmonary flow velocity, m/s ^t^	1.00 ± 0.19; 1.00 (0.63–1.40)	0.92 ± 0.17; 0.90 (0.60–1.30)	0.076	*d* = 0.455
Tricuspid annular plane systolic excursion (TAPSE), mm ^t^	20.05 ± 3.65; 20.00 (14.00–28.00)	21.61 ± 4.05; 21.00 (14.00–29.00)	0.093	*d* = −0.405
Systolic pulmonary artery pressure (sPAP), mmHg ^z^	27.54 ± 8.34; 27.00 (14.00–50.00)	24.12 ± 4.13; 25.00 (15.00–30.00)	0.058	*r* = 0.227
Left ventricular mass, g ^z^	201.54 ± 78.78; 185.00 (100.60–448.71)	195.18 ± 54.50; 187.28 (77.38–337.90)	0.930	*r* = 0.010
Left ventricular mass index (LVMI)	111.03 ± 38.04; 105.83 (60.52–226.50)	102.83 ± 25.98; 95.52 (46.84–151.75)	0.467	*r* = 0.089

Note: Data are presented as *n* (%) for categorical variables and as mean ± standard deviation and median (minimum–maximum) for continuous variables. Categorical variables were compared using Pearson’s chi-square test or Fisher’s exact test, as appropriate. Normally distributed continuous variables were compared using the independent-samples *t*-test, and non-normally distributed continuous variables were compared using the Mann–Whitney *U* test. ^t^ Independent-samples *t*-test; effect sizes were expressed as Cohen’s *d*. ^z^ Mann–Whitney *U* test; effect sizes were expressed as *r* = |*Z*|/√*N*. For Cohen’s *d*, values of 0.20, 0.50, and 0.80 were considered small, moderate, and large effects, respectively; for *r*, values of 0.10, 0.30, and 0.50 were considered small, moderate, and large effects, respectively. Echocardiographic data were available for all 73 patients, with no missing echocardiographic data. Bold values indicate statistically significant differences (* *p* < 0.05).

**Table 4:** Correlation Analysis of Coffee Consumption Status and Monthly Coffee Consumption with Selected Clinical, Biochemical, and Echocardiographic Variables.

Variable	Coffee Consumption Status ^1^		Coffee Consumption Amount (g/Month) ^2^	
	*r*	*p*	*r*	*p*
Demographic, clinical, and lifestyle characteristics				
Sex (female)	−0.036	0.760	−0.149	0.394
Presence of diabetes mellitus	−0.160	0.176	−0.018	0.919
Presence of hypertension	−0.122	0.305	0.257	0.136
Smoking status	**0.313**	0.007 *	**0.380**	0.025 *
Presence of mitral regurgitation	0.038	0.747	**0.549**	<0.001 *
Presence of aortic regurgitation	−0.007	0.954	0.098	0.577
Presence of tricuspid regurgitation	0.043	0.717	−0.049	0.780
Presence of increased left ventricular mass index	−0.155	0.189	−0.074	0.674
Age (years)	−0.070	0.558	**0.442**	0.008 *
Disease duration (months)	0.008	0.943	0.311	0.069
Follow-up duration (months)	0.122	0.306	**0.423**	0.011 *
Smoking exposure (pack-years)	0.329	0.071	−0.180	0.435
Total daily fluid intake (L/day)	0.100	0.400	0.107	0.539
Anthropometric characteristics				
Body mass index (kg/m^2^)	0.016	0.896	−0.105	0.560
Total body water (kg)	0.176	0.154	−0.059	0.742
Total body water (%)	0.042	0.738	0.090	0.618
Body surface area (m^2^)	0.196	0.112	−0.158	0.380
Serum copeptin level and laboratory parameters				
Serum copeptin level (ng/L)	**−0.319**	0.012 *	**0.393**	0.024 *
Glucose (mg/dL)	−0.009	0.939	0.224	0.196
Urea (mg/dL)	0.051	0.666	0.253	0.142
Creatinine (mg/dL)	0.113	0.343	0.175	0.314
eGFR (mL/min/1.73 m^2^)	−0.079	0.504	−0.237	0.170
Uric acid (mg/dL)	−0.007	0.952	−0.023	0.897
Calcium (mg/dL)	−0.065	0.588	−0.095	0.588
Sodium (mmol/L)	−0.110	0.354	0.160	0.357
Potassium (mmol/L)	−0.133	0.262	0.328	0.054
Phosphate (mg/dL)	−0.078	0.511	−0.066	0.704
Parathyroid hormone (pg/mL)	0.052	0.662	−0.037	0.835
Leukocyte count (×10^3^/µL)	0.052	0.664	0.108	0.537
Hemoglobin (g/dL)	0.028	0.815	0.108	0.538
Hematocrit (%)	0.066	0.580	0.073	0.676
Platelet count (×10^3^/µL)	−0.152	0.201	0.135	0.440
Spot urine albumin-to-creatinine ratio (mg/g)	0.054	0.650	0.068	0.696
Echocardiographic parameters				
Left ventricular end-diastolic diameter (LVDd, cm)	0.073	0.539	−0.075	0.669
Left ventricular end-systolic diameter (LVSd, cm)	0.012	0.917	−0.066	0.707
Interventricular septal thickness (IVST, cm)	−0.109	0.361	−0.050	0.774
Left ventricular posterior wall thickness (PWT, cm)	0.018	0.881	−0.061	0.726
Left ventricular ejection fraction (LVEF, %)	−0.003	0.978	0.075	0.670
Fractional shortening (FS, %)	−0.036	0.763	0.128	0.463
Left ventricular end-diastolic volume (LVEDV, mL)	0.012	0.923	0.066	0.710
Left ventricular end-systolic volume (LVESV, mL)	−0.091	0.451	0.085	0.634
Aortic root diameter (cm)	−0.043	0.721	0.148	0.396
Ascending aortic diameter (AscAo, cm)	0.003	0.984	0.181	0.357
Left atrial diameter (LAD, cm)	−0.002	0.987	0.046	0.794
Mitral early diastolic flow velocity (E, cm/s)	−0.087	0.478	0.089	0.622
Mitral late diastolic flow velocity (A, cm/s)	−0.100	0.409	**0.352**	0.044 *
Mitral annular early diastolic tissue velocity (e′, cm/s)	**0.408**	<0.001 *	0.044	0.807
Mitral E/e′ ratio	**−0.414**	<0.001 *	−0.058	0.752
Aortic flow velocity (m/s)	0.165	0.233	0.158	0.431
Pulmonary flow velocity (m/s)	−0.212	0.095	−0.128	0.492
Tricuspid annular plane systolic excursion (TAPSE, mm)	0.186	0.120	−0.115	0.525
Systolic pulmonary artery pressure (sPAP, mmHg)	−0.229	0.057	0.082	0.650
Left ventricular mass (g)	0.010	0.930	−0.133	0.445
Left ventricular mass index (g/m^2^)	−0.090	0.471	−0.019	0.916

Note: Relationships were assessed using Spearman’s rank correlation analysis. *r*: Spearman’s correlation coefficient. ^1^ Coffee consumption status was coded as 0 = no and 1 = yes, and correlations were examined in the entire study population. ^2^ Analyses of monthly coffee consumption (g/month) were performed only among coffee consumers. Sex was coded as 0 = male and 1 = female; other binary variables were coded as 0 = no and 1 = yes. Missing data were excluded separately for each pair of variables. Bold values indicate statistically significant correlations (* *p* < 0.05). The findings were considered exploratory.

**Table 5:** Log-Linear Regression Analysis of the Association Between Monthly Coffee Consumption Amount and Serum Copeptin Levels in Coffee-Consuming Patients with Autosomal Dominant Polycystic Kidney Disease.

Model	Variable	B	SE	β	t	*p*	95% CI for B
Unadjusted	Constant	5.024	0.274	—	18.369	<0.001 *	4.466–5.582
	LN(coffee consumption amount)	**0.154**	**0.062**	**0.408**	**2.490**	**0.018** *	**0.028–0.280**
Adjusted	Constant	5.524	0.535	—	10.326	<0.001 *	4.428–6.620
	LN(coffee consumption amount)	0.133	0.074	0.353	1.807	0.082	−0.018–0.284
	Age (years)	−0.004	0.008	−0.126	−0.541	0.592	−0.020–0.011
	Smoking status	0.138	0.154	0.158	0.892	0.380	−0.179–0.454
	eGFR (mL/min/1.73 m^2^)	−0.004	0.003	−0.272	−1.260	0.218	−0.010–0.002

Model summary: Unadjusted model: *n* = 33; R^2^ = 0.167; adjusted R^2^ = 0.140; F(1,31) = 6.201; *p* = 0.018. Adjusted model: *n* = 33; R^2^ = 0.237; adjusted R^2^ = 0.129; F(4,28) = 2.180; *p* = 0.097. Note: The dependent variable was the natural logarithm of serum copeptin levels [LN(copeptin)]. Monthly coffee consumption amount (g/month) was natural log-transformed. Analyses were performed only among 33 coffee-consuming patients with complete data for all model variables. Age, smoking status, and eGFR were simultaneously included in the adjusted model. Smoking status was coded as 0 = No and 1 = Yes. B: unstandardized regression coefficient; SE: standard error; β: standardized regression coefficient; CI: confidence interval. Bold values indicate statistically significant results (* *p* < 0.05).

**Table 6:** Multivariable Linear Regression Analysis of the Association Between Coffee Consumption and Serum Copeptin Levels Adjusted for Smoking Status and Other Covariates.

Independent Variable	B	SE	β	t	*p*	95% CI for B	Tolerance	VIF
Constant	5.888	0.477	—	12.357	<0.001 *	4.933–6.843	—	—
Age (years)	0.004	0.005	0.129	0.730	0.468	−0.007–0.015	0.499	2.003
Sex ^1^	−0.073	0.131	−0.079	−0.559	0.578	−0.335–0.189	0.776	1.288
Coffee consumption ^2^	−0.263	0.129	−0.286	−2.031	**0.047** *	−0.522–−0.004	0.781	1.280
Smoking status ^3^	0.061	0.136	0.066	0.446	0.657	−0.213–0.334	0.717	1.394
Total daily fluid intake (L/day)	0.028	0.093	0.039	0.297	0.768	−0.158–0.214	0.876	1.141
eGFR (mL/min/1.73 m^2^)	−0.002	0.003	−0.160	−0.914	0.365	−0.008–0.003	0.505	1.978

Model summary: R = 0.386; R^2^ = 0.149; adjusted R^2^ = 0.056; F(6,55) = 1.606; *p* = 0.163. Note: The dependent variable was the natural logarithm of serum copeptin [ln(copeptin)]. The analysis included 62 patients with complete data for all variables included in the model. ^1^ Sex: 0 = male, 1 = female. ^2^ Coffee consumption: 0 = no, 1 = yes. ^3^ Smoking status: 0 = no, 1 = yes. B: unstandardized regression coefficient; SE: standard error; β: standardized regression coefficient; CI: confidence interval; VIF: variance inflation factor. Bold values indicate statistically significant results (* *p* < 0.05).

**Table 7:** Multivariable Linear Regression Sensitivity Analysis of the Association Between Coffee Consumption and Serum Copeptin Levels Using Pack-Years as a Measure of Smoking Exposure.

Independent Variable	B	SE	β	t	*p*	95% CI for B	VIF
Constant	5.920	0.472	—	12.532	<0.001 *	4.974–6.867	—
Age (years)	0.004	0.005	0.126	0.706	0.483	−0.007–0.015	2.065
Sex (female)	−0.088	0.130	−0.095	−0.673	0.504	−0.349–0.173	1.279
Coffee consumption	−0.248	0.131	−0.270	−1.890	0.064	−0.510–0.015	1.310
Smoking exposure (pack-years)	0.001	0.006	0.020	0.131	0.896	−0.010–0.012	1.437
Total daily fluid intake (L/day)	0.026	0.093	0.038	0.285	0.777	−0.160–0.213	1.141
eGFR (mL/min/1.73 m^2^)	−0.002	0.003	−0.164	−0.940	0.351	−0.008–0.003	1.972

Model summary: R = 0.382; R^2^ = 0.146; adjusted R^2^ = 0.053; F(6,55) = 1.571; *p* = 0.173. Note: The dependent variable was the natural logarithm of serum copeptin [ln(copeptin)]. The analysis included 62 patients with complete data for all variables included in the model. Coffee consumption was coded as 0 = no, 1 = yes; sex was coded as 0 = male, 1 = female. For participants who reported no smoking and had blank pack-year records, smoking exposure was assigned a value of 0 pack-years; recorded positive pack-year values were retained. All independent variables were entered simultaneously into the model. B: unstandardized regression coefficient; SE: standard error; β: standardized regression coefficient; CI: confidence interval; VIF: variance inflation factor. Bold values indicate statistically significant results (* *p* < 0.05).

**Table 8:** Multivariable Linear Regression Analyses of the Associations of Coffee Consumption with Mitral Annular e′ Velocity and Mitral E/e′ Ratio.

Dependent Variable	Independent Variable	B	SE	β	t	*p*	95% CI for B	Tolerance	VIF
Mitral annular e′ velocity (cm/s)	Constant	7.989	2.387	—	3.346	0.001 *	3.218 to 12.759	—	—
	Age (years)	−0.066	0.028	−0.324	−2.361	0.021 *	−0.122 to −0.010	0.594	1.683
	Sex ^1^	1.380	0.742	0.214	1.860	0.067	−0.102 to 2.863	0.843	1.187
	Coffee consumption ^2^	2.531	0.726	0.395	3.488	<0.001 *	1.081 to 3.981	0.872	1.147
	Smoking ^3^	0.832	0.801	0.128	1.039	0.303	−0.768 to 2.432	0.735	1.360
	Hypertension ^4^	1.145	0.725	0.179	1.579	0.119	−0.304 to 2.594	0.870	1.149
	eGFR (mL/min/1.73 m^2^)	−0.007	0.014	−0.066	−0.481	0.632	−0.034 to 0.021	0.588	1.700
Mitral E/e′ ratio	Constant	10.818	5.300	—	2.041	0.046 *	0.212 to 21.425	—	—
	Age (years)	0.102	0.061	0.247	1.665	0.101	−0.021 to 0.224	0.604	1.655
	Sex ^1^	−2.519	1.674	−0.189	−1.504	0.138	−5.870 to 0.832	0.841	1.190
	Coffee consumption ^2^	−4.548	1.618	−0.347	−2.811	0.007 *	−7.786 to −1.310	0.871	1.148
	Smoking ^3^	−0.940	1.782	−0.071	−0.527	0.600	−4.506 to 2.626	0.742	1.347
	Hypertension ^4^	−3.404	1.625	−0.260	−2.095	0.040 *	−6.655 to −0.153	0.864	1.158
	eGFR (mL/min/1.73 m^2^)	0.013	0.030	0.064	0.430	0.668	−0.048 to 0.074	0.602	1.661

Model summary: Mitral annular e′ velocity model: *n* = 70; R = 0.543; R^2^ = 0.295; adjusted R^2^ = 0.227; F(6,63) = 4.384; *p* < 0.001. Mitral E/e′ ratio model: *n* = 66; R = 0.464; R^2^ = 0.216; adjusted R^2^ = 0.136; F(6,59) = 2.702; *p* = 0.022. Notes: Mitral annular e′ velocity and mitral E/e′ ratio were evaluated as dependent variables in separate models. Age, sex, coffee consumption, smoking, hypertension, and eGFR were entered simultaneously using the Enter method. ^1^ Sex: 0 = male, 1 = female. ^2^ Coffee consumption: 0 = no, 1 = yes. ^3^ Smoking: 0 = no, 1 = yes. ^4^ Hypertension: 0 = no, 1 = yes. B, unstandardized regression coefficient; SE, standard error; β, standardized regression coefficient; CI, confidence interval; eGFR, estimated glomerular filtration rate; VIF, variance inflation factor. Bold values indicate statistically significant results (* *p* < 0.05).

## Data Availability

Data are unavailable due to privacy or ethical restrictions.
